# Prediction of short-term mortality in acute heart failure patients using minimal electronic health record data

**DOI:** 10.1186/s13040-021-00255-w

**Published:** 2021-03-31

**Authors:** Ashwath Radhachandran, Anurag Garikipati, Nicole S. Zelin, Emily Pellegrini, Sina Ghandian, Jacob Calvert, Jana Hoffman, Qingqing Mao, Ritankar Das

**Affiliations:** Dascena, Inc, 12333 Sowden Rd Ste B PMB 65148, Houston, TX 77080-2059 USA

**Keywords:** Acute heart failure, Mortality, Machine learning, Prediction, Clinical decision support

## Abstract

**Background:**

Acute heart failure (AHF) is associated with significant morbidity and mortality. Effective patient risk stratification is essential to guiding hospitalization decisions and the clinical management of AHF. Clinical decision support systems can be used to improve predictions of mortality made in emergency care settings for the purpose of AHF risk stratification. In this study, several models for the prediction of seven-day mortality among AHF patients were developed by applying machine learning techniques to retrospective patient data from 236,275 total emergency department (ED) encounters, 1881 of which were considered positive for AHF and were used for model training and testing. The models used varying subsets of age, sex, vital signs, and laboratory values. Model performance was compared to the Emergency Heart Failure Mortality Risk Grade (EHMRG) model, a commonly used system for prediction of seven-day mortality in the ED with similar (or, in some cases, more extensive) inputs. Model performance was assessed in terms of area under the receiver operating characteristic curve (AUROC), sensitivity, and specificity.

**Results:**

When trained and tested on a large academic dataset, the best-performing model and EHMRG demonstrated test set AUROCs of 0.84 and 0.78, respectively, for prediction of seven-day mortality. Given only measurements of respiratory rate, temperature, mean arterial pressure, and FiO_2_, one model produced a test set AUROC of 0.83. Neither a logistic regression comparator nor a simple decision tree outperformed EHMRG.

**Conclusions:**

A model using only the measurements of four clinical variables outperforms EHMRG in the prediction of seven-day mortality in AHF. With these inputs, the model could not be replaced by logistic regression or reduced to a simple decision tree without significant performance loss. In ED settings, this minimal-input risk stratification tool may assist clinicians in making critical decisions about patient disposition by providing early and accurate insights into individual patient’s risk profiles.

**Supplementary Information:**

The online version contains supplementary material available at 10.1186/s13040-021-00255-w.

## Introduction

Acute heart failure (AHF) refers to new or worsening signs and symptoms of heart failure leading to unscheduled medical care or hospitalization [1]. The condition can arise de novo or as an acute decompensation of pre-existing chronic heart failure [1]. AHF is among the most frequent causes of hospitalization in the United States and represents a growing healthcare burden [2, 3]. Expenditures for AHF in the United States approach $39 billion per year, and are expected to almost double by 2030 [4, 5]. An episode of AHF can herald the onset of rapidly progressive and ultimately fatal disease: up to half of heart failure patients die within 5 years of first diagnosis [6–9].

Initial evaluation and triage of AHF patients is often performed in the emergency department (ED). Based on this initial clinical assessment, patients may be discharged for outpatient management or admitted for more intensive care. Accurate risk stratification of AHF patients is important for optimizing outcomes, as it may be used to guide hospitalization decisions and clinical management [10]. Estimating near-term mortality has been the cornerstone of risk stratification in AHF [11]. However, even skilled clinicians may incorrectly prognosticate risk of death in heart failure patients [12, 13]. The heterogeneity of the underlying disease process, particularly in de novo AHF, presents a prognostic challenge, since the clinical course of patients with different etiologies of AHF may vary [14–16]. Risk scoring systems may be used to improve predictions made in the ED context about mortality in AHF patients beyond clinical judgment alone [10, 12]. Numerous risk scores have been developed, mostly using conventional multivariable statistical modeling [17].

Machine learning (ML), a form of artificial intelligence, provides an opportunity to improve upon predictions made with such risk scoring systems. Machine learning algorithms (MLAs) can make clinically relevant predictions about the occurrence of future events based on data available within the electronic health record (EHR) [18]. Many conventional AHF mortality risk scores utilize “one size fits all” scoring rules and are frequently derived from populations non-representative of the spectrum of patients seen in real world clinical practice [10]. In contrast, MLAs provide individualized patient risk assessments and may be trained on data from diverse, real-life populations [19]. MLAs can utilize large amounts of data available in the EHR in a manner that is not feasible for tools which require manual scoring, integrating both dynamic changes within, and complex relationships between, variables into making predictions [20]. MLAs have been shown to outperform existing mortality prediction approaches in other areas of cardiovascular medicine, supporting their potential utility for predicting mortality in AHF patients [17, 21, 22]. Indeed, foundational research has illustrated that various types of MLAs can outperform conventional risk scoring systems at predicting mortality in AHF patients [23, 24]. We hypothesized that a machine learning based classifier using minimal inputs may be an effective tool for risk stratification in AHF, and may improve upon the performance of existing mortality prediction tools. This work describes the development and retrospective validation of a gradient boosted decision trees model to predict the likelihood of seven-day mortality in AHF patients based on minimal EHR data during initial patient evaluation in the ED.

## Results

**Fig. 1:**
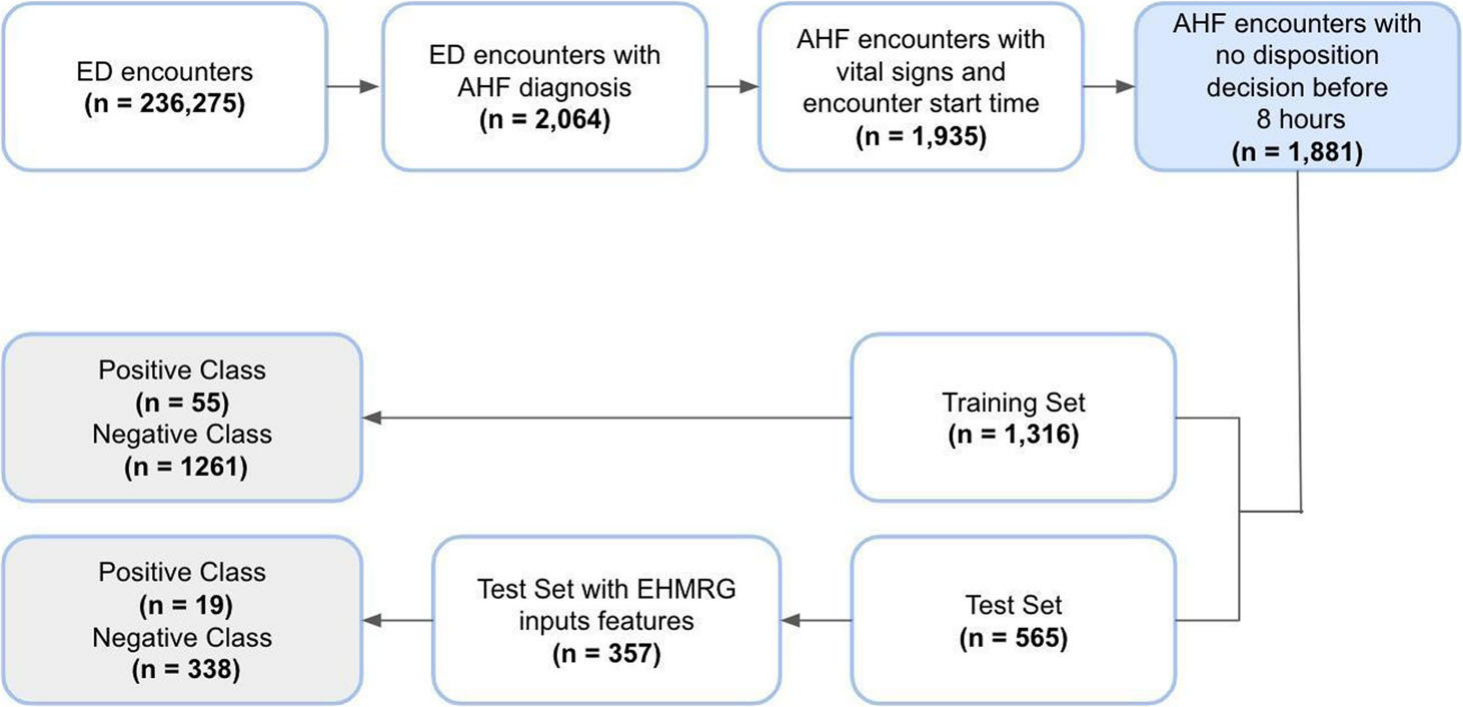
Patient encounter inclusion diagram. Abbreviations used: acute heart failure (AHF); Emergency Department (ED); Emergency Heart Failure Mortality Risk Grade (EHMRG)

A total of 236,275 ED patient encounters were available in our dataset. Of these encounters, 1881 were associated with an AHF diagnosis, had complete chart data and were present in the ED at eight hours (Fig. 1). For hold-out testing of the models, a total of 357 patients were included, 19 of whom had a positive mortality event as defined by our gold standard (Fig. 1).

Demographic data for the combined training and testing data are presented in (Table 1). AHF patients with a seven-day mortality event were, on average, more likely to be elderly (age > 80 years), more likely to have cancer and dementia, and less likely to have hypertension (Table 1). Median age for the positive class was 82 years (interquartile range (IQR): 73, 90), and median age among the negative class was 74 (IQR: 61, 85).

**Table 1:** Demographics of the combined data used to train and test models for the prediction of seven-day mortality in acute heart failure patients presenting to the Emergency Department. Differences between the positive and negative class were evaluated for significance with a two proportions z-test

	AHF patients who die within seven days N (%)	AHF patients who do not die within seven days N (%)	*P* value
Age			
< 30	0 (0.00)	25 (1.56)	0.2785
30–49	2 (2.70)	153 (9.57)	0.0464
50–59	7 (9.46)	205 (12.82)	0.3955
60–69	8 (10.81)	315 (19.70)	0.0582
70–80	17 (22.97)	311 (19.45)	0.4555
80+	40 (54.05)	590 (36.90)	0.0029
Sex			
Female	33 (44.6)	664 (41.5)	0.601
Male	41 (55.4)	935 (58.5)	0.601
Race			
American Indian or Alaska Native	0 (0)	1 (0.09)	0.8296
Asian	26 (35.14)	256 (22.72)	<.0001
Black or African American	8 (10.81)	204 (18.1)	0.6225
Native Hawaiian or Other Pacific Islander	4 (5.41)	31 (2.75)	0.0416
Other	10 (13.51)	149 (13.22)	0.2290
Unknown/Declined	1 (1.35)	20 (1.77)	0.9394
Ethnicity			
Hispanic or Latino	5 (6.76)	93 (8.25)	0.7362
Not Hispanic or Latino	65 (87.84)	1013 (89.88)	<.0001
Unknown/Declined	4 (5.41)	21 (1.86)	0.0046
Medical Comorbidities			
Dyslipidemia	21 (28.38)	590 (36.9)	0.1367
Diabetes Mellitus	28 (37.84)	703 (43.96)	0.2989
Hypertension	46 (62.16)	1223 (76.49)	0.0049
Peripheral Vascular Disease	4 (5.41)	138 (8.63)	0.3305
Atrial Fibrillation	38 (51.35)	757 (47.34)	0.4996
Chronic Kidney Disease	41 (55.41)	839 (52.47)	0.6211
Hepatic Cirrhosis	4 (5.41)	76 (4.75)	0.7971
Chronic Obstructive Pulmonary Disease	22 (29.73)	399 (24.95)	0.3546
Cancer	21 (28.38)	145 (9.07)	<.0001
Dementia	12 (16.22)	129 (8.07)	0.0136
Depression	6 (8.11)	176 (11.01)	0.4337
History of TIA or Ischemic Stroke	2 (2.7)	54 (3.38)	0.7525
History of MI	12 (16.22)	261 (16.32)	0.9807

Four models were trained: a 33-feature XGBoost model (33F) and a logistic regression (LR) model trained using all inputs in Supplementary Table 1, a five-feature XGBoost model (Top5F) and a five-feature decision tree (DT). From a variety of inputs provided based on a priori relevance to AHF mortality prediction, XGBoost selected the 33 features most useful to making predictions; these inputs were then used by the 33F model and the LR model. A SHAP analysis of the 33F XGBoost model revealed the input features that XGBoost determined to be the most important in making predictions. The top five most important features were then provided as inputs to the Top5F XGBoost model. The Top5F and DT models used the same five features as inputs. These four models were tested alongside EHMRG for the prediction of the seven-day mortality gold standard, where predictions were made at the eighth hour of each stay.

**Fig. 2:**
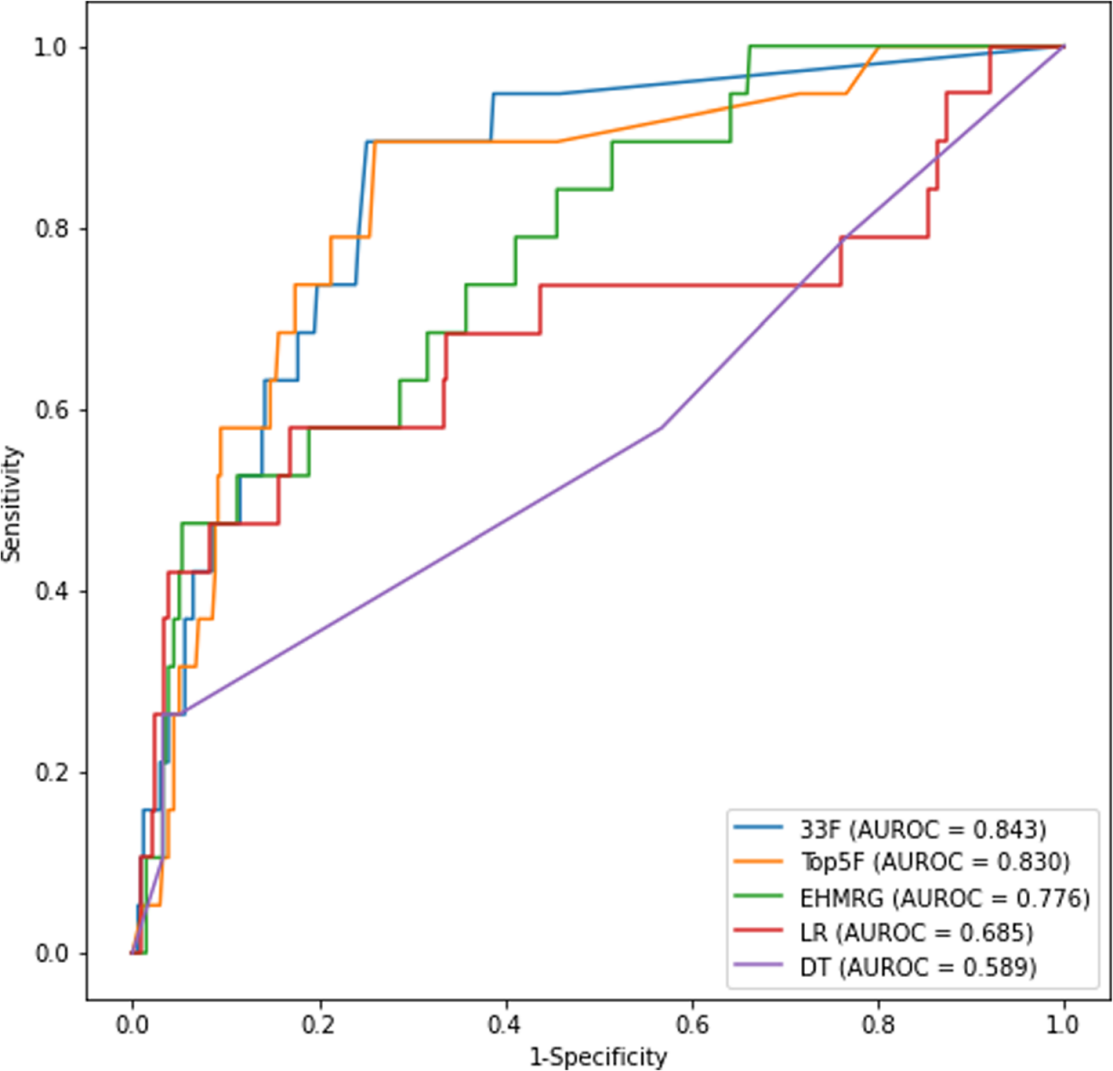
Receiver operating characteristic (ROC) curves for the prediction of seven-day mortality among acute heart failure patients for gradient-boosted decision trees models with 33 and five features (33F and Top5F, respectively), the Emergency Heart Failure Mortality Risk Grade (EHMRG) model, a logistic regression (LR) model, and a single decision tree (DT) with the same five features as Top5F

The receiver operating characteristic (ROC) curves for the models on the hold-out test set are presented in Fig. 2, demonstrating that 33F and Top5F exhibit greater sensitivity than EHMRG across a range of specificities, while DT and LR do not. Similarly, models 33F and Top5F achieved a greater area under the receiver operating characteristic (AUROC) curve than EHMRG, DT, and LR (Table 2). Operating points for the models were selected to maximize sensitivity subject to specificity. For these operating points, 33F and Top5F outperformed the EHMRG on all evaluated performance metrics (Table 2).

**Table 2:** Performance metrics of the gradient-boosted decision trees models with 33 and five features (33F and Top5F, respectively), the Emergency Heart Failure Mortality Risk Grade (EHMRG) model, a single decision tree (DT) with the same five features as Top5F, and a logistic regression (LR) model for the prediction of seven-day mortality

	33F	Top5F	EHMRG	DT	LR
**AUROC**	**0.843**	0.830	0.776	0.589	0.685
**Sensitivity**	**0.895**	**0.895**	0.684	0.211	0.579
**Specificity**	0.749	0.618	0.642	**0.967**	0.722
**LR+**	3.558	2.344	1.911	**6.469**	2.082
**LR-**	**0.141**	0.170	0.492	0.816	0.583
**DOR**	**25.300**	13.771	3.886	7.927	3.569

**Fig. 3:**
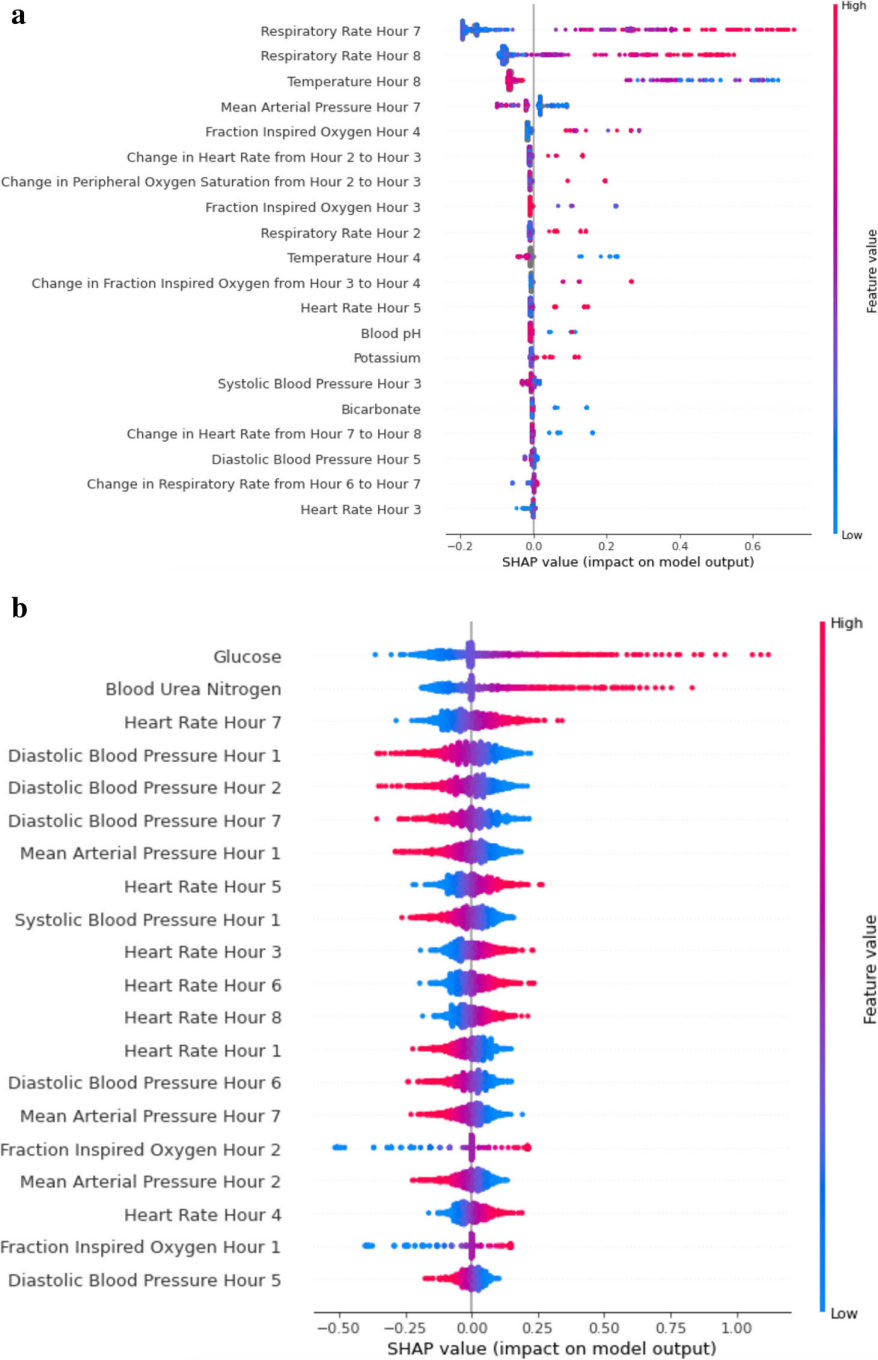
Feature correlations and distribution of feature importance for (**a**) the 33-feature gradient-boosted decision trees model (33F) and (**b**) the logistic regression (LR) model. Model input variables are ranked in descending order of feature importance. Red is indicative of a high feature value and blue is indicative of a low feature value. Points to the right of the line of neutral contribution resulted in a higher score; points to the left of this line resulted in a lower score

The two most recent (i.e., at the seventh and eighth hour of the stay) measurements of respiratory rate, and single measurements of temperature, mean arterial pressure, and FiO_2_ (the features of model Top5F), were the most important features for 33F, as measured by their SHAP values (Fig. 3). Laboratory test results were also important for 33F. For LR, serum glucose and blood urea nitrogen were the most important features. Repeated blood pressure and heart rate measurements had relatively high SHAP values, suggesting the importance of dynamic measurements for LR. Feature correlations and distribution of feature importance for each patient encounter for each model are presented in Fig. 3.

## Discussion

### Key findings

Accurate AHF patient risk stratification in the ED is critical to informing clinical management decisions, with implications both for patient outcomes and healthcare costs. In this study, we describe the development of models which can accurately predict seven-day mortality among AHF patients in the ED setting using only a subset of age, sex, and a limited collection of common clinical and laboratory measurements as inputs (Supplementary Table 1). In particular, one model approximated the best performance using a total of only five observations (in total) of four clinical variables–respiratory rate, temperature, mean arterial pressure, and FiO_2_.

The best-performing model used only 33 features derived from 20 clinical variables. Model 33F achieved a sensitivity of 89.5% and specificity of 74.9%, with an AUROC of 0.843 (Fig. 2), demonstrating a low false negative rate while accurately discriminating between low- and high-risk patient groups. Cardiovascular risk prediction models that obtain AUROCs above 0.8 are generally considered to have strong discriminatory performance [25]. Notably, 33F outperformed the EHMRG on all performance metrics (Table 2). Previous research has shown that the EHMRG can perform with high discrimination (e.g. AUROC > 0.7) in ED populations [26, 27], underscoring that an MLA can improve upon even well-performing clinical prediction tools developed using conventional statistical methods.

Using only five measurements, model Top5F effectively matched the AUROC of model 33F and offered an operating point with the same sensitivity but lower specificity (Fig. 2, Table 2). Model Top5F may be preferable to model 33F given the benefits provided by the use of fewer inputs. However, as predictive models of acute conditions sometimes use thousands of features [28], Top5F and 33F can both be considered to use a minimal set of inputs.

### Justification for model architectures

There are many advantages to using a minimal set of commonly available inputs for MLAs [29, 30]. First, using fewer measurements lessens the risk of overfitting the data and avoids poor generalization of model performance. Second, by using inputs which are measured for most patients (and measured frequently), one increases the chance of model predictions being made on the basis of complete and up-to-date information. Third, by using few measurements, one greatly reduces the burden of model integration with the EHR during prospective implementation. Fourth, models which use a few, easy-to-collect inputs may be adapted to limited resource settings.

To determine if a structurally simpler model could be substituted for Top5F while outperforming EHMRG, we experimented with two other models–a logistic regression using the same inputs as 33F, and a single decision tree using the same five inputs as Top5F. A similar approach was used to produce a “clinically portable” decision tree model for COVID-19 mortality prediction, to be used in place of a corresponding gradient-boosted decision trees model [31]. We found that a structurally simpler model could not be substituted for Top5F without significant performance losses and a failure to exceed the benchmark provided by EHMRG (Fig. 2, Table 2). Specifically, the structural simplicity and limited depth of the simple DT prevent this model from effectively using the limited input features, likely contributing to the poor sensitivity and AUROC. By its nature, a simple DT is a weak learner relative to an ensemble learning approach like XGBoost, which successively learns from a collection of decision trees to ultimately create a more powerful model. XGBoost creates this ensemble of decision trees by iteratively building trees which perform better on misclassified examples.

Among the many MLAs suitable for creating mortality prediction models, we chose the XGBoost algorithm because of the ease with which it handles missing or null values [32]. Given the high levels of “missingness” within EHR data - due to variability in data acquisition and recording habits in the live clinical environment - this quality is attractive, even when using common and frequently measured clinical variables [33]. Boosted regression classifiers generally, and XGBoost specifically, perform as well or better than other machine learning approaches in predicting mortality among heart failure patients [34–36]. XGBoost architectures are appropriate for training from minimal feature sets because they are able to learn adaptively and can utilize dynamic data. While EHMRG uses a predetermined set of validated inputs, XGBoost models can use these inputs or a wider range of inputs, testing and selecting the best inputs to make accurate predictions. In addition, the EHMRG only uses static features which cannot account for changes (e.g. in laboratory measurements or vital signs) during an ED encounter. These differences likely enabled the XGBoost models to attain higher performance compared to EHMRG (Table 2).

### Providing context: alternative risk scores and prediction systems

The most important features used by the models were consistent with prognostic factors reported in the literature. For example, increased respiratory rate in the ED has been linked to ICU admission, mechanical ventilation and death in AHF patients [37]. Hemodynamic instability has also been associated with a poor prognosis for AHF patients, including death post-discharge [38]. The importance of repeated vital sign measurements at different time points–as well as the changes in vital signs measurements–in generating predictions highlights the value of a machine learning-based tool which can effectively process dynamic trends in data [17].

Increased attention to the potential clinical and economic benefits of effective AHF risk stratification has led to the development of risk scores for use in the ED [39], including the EHMRG [16]. However, many extant risk stratification tools were derived from populations not representative of the heterogeneous spectrum of real world ED patients, or have only been validated for risk evaluation in certain sub-types of AHF [10]. Few risk tools have been developed with clear, robust methods for adjusting prediction to account for missing input data [17]. The effectiveness of conventional risk scores in supporting clinical decision making may be hindered by decision rule complexity and by the absence of standardized use across providers and healthcare systems [40–42]. Further, conventional scores evaluate the risk of a cohort of patients with similar characteristics, and do not provide an individualized assessment of risk for each patient [10].

MLAs have been developed to accomplish a variety of tasks in the management of heart failure, including diagnosis, classification of disease type, prediction of outcomes, and predicting response to treatment [43–46]. A rapidly growing body of work has focused on the use of machine learning for prediction of mortality in patients with AHF [23, 47, 48, 49]. For example, Kwon et al. developed and validated a deep neural network algorithm that predicted in-hospital, 1- and 3- year mortality among AHF patients more accurately than both the conventional Guidelines-Heart Failure (GWTG-HF) risk score [48] and other machine learning approaches [23]. The deep neural network developed by Kwon et al. obtained AUROCs of 0.880, 0.782, and 0.813 for prediction of in-hospital, 1 year, and 3 year mortality, respectively. Models 33F and Top5F achieved comparable results (AUROCs of 0.843 and 0.830), without requiring the abundance of input variables and large-scale training data neural networks require [50].

Adler et al. developed an MLA to discriminate between heart failure patients at high and low risk of death [47] and Awan et al. used a battery of similar machine learning methods to predict risk of readmission or death in elderly heart failure patients [48]. However, the models developed in these studies were not specifically designed and trained for use in ED settings. It is important that models intended to guide patient disposition planning are developed on targeted patient populations who present to the ED, as opposed to being developed on broader hospitalized populations [16]. While some MLAs designed to predict mortality in heart failure have not been validated outside of training data [17], the XGBoost model in this study demonstrated strong performance on a hold-out test set, suggesting that it was not overfit in training and can successfully make predictions on other data.

Both conventional risk models and MLAs have been developed which leverage B-type natriuretic peptide (BNP) or N-terminal prohormone BNP (NT-proBNP) as inputs [36, 51, 52]. These data were not available within our dataset, and so could not be incorporated as input features. BNP and NT-proBNP can be useful in diagnostic evaluation and have prognostic value, including for mortality prediction [9, 53–56]. Models 33F and Top5F were able to perform well without these laboratory results, and adaptations of these models to clinical datasets in which these results are available have the potential to perform better.

### Limitations

This study has several limitations. Our inclusion criteria resulted in a relatively small positive class size, which may limit generalizability of results. Our dataset also did not include information on home metolazone use or whether patients arrived at the ED via emergency medical services. Without these inputs, the EHMRG could not be calculated to yield scores comparable to the risk thresholds described in the literature. However, metolazone use has previously been identified as contributing minimally to final EHMRG risk categorizations [57]. In addition, our approach to selecting a threshold intentionally optimized the performance of the adjusted EHMRG. The AUROC of the adjusted EHMRG in our study is comparable with reported AUROCs of the unadjusted EHMRG in the literature [26, 27], suggesting that the performance of the EHMRG was preserved and remains a suitable comparison for the performance of the models. Further, because this is a retrospective study, we were unable to determine the performance of the AHF prediction algorithms in a prospective clinical setting. Prospective validation is required to determine how clinicians may respond to predictions of mortality among AHF patients, as well as to determine whether predictions can affect patient outcomes or resource allocation.

## Conclusions

A model using only four clinical variables outperforms EHMRG in the prediction of seven-day mortality of AHF patients in the ED. This tool may assist clinicians in making critical decisions about patient disposition by providing early and accurate insights into individual patient’s risk profiles.

## Materials and methods

### Data processing

The MLA development workflow, including data processing, is outlined in Supplementary Fig. 1. Patient data were extracted from the EHR of a large academic medical center. We included data extracted from patients between 2011 and 2015 who were assessed in the ED. These data included patient demographics, medical diagnoses from prior encounters, vital signs, and laboratory data. Data were de-identified in compliance with the Health Insurance Portability and Accountability Act (HIPAA) and were collected passively. Since data were de-identified and collected retrospectively, this study was considered non-human subjects research and did not require Institutional Review Board approval.

We only included data from patients with ED encounters for AHF and complete chart data. ED encounters for AHF were identified by the presence of International Classification of Diseases, Tenth Revision (ICD-10) codes for acute heart failure in the inpatient chart (Supplementary Table 2). No data were included for ED encounters not associated with an AHF diagnosis. Chart data were considered to be incomplete if it did not contain any vital sign measurements or if it did not contain a start time for the ED encounter.

Seven-day mortality among AHF patients was the primary endpoint. Based on the desired functionality of this MLA–as a clinical decision tool which physicians might use when making clinical management and patient disposition decisions–it was necessary to select a prediction time at which the majority of AHF patients had not already been admitted or discharged from the ED. For the majority of AHF patients with a documented disposition time in our dataset (77.5%), admission or discharge occurred after 8 h into the ED encounter. Therefore, the MLA was designed to generate predictions 8 h after the start of the ED encounter, and all patients with a documented disposition decision prior to 8 h were excluded from analysis.

Algorithm scores were computed using patient demographics, clinical and laboratory measurements, and medical history extracted from the EHR (Supplementary Table 2). Vital sign measurements were averaged across each hour to yield a single mean vital sign measurement at each hour. The change in value between the hourly average vital sign measurements was also used as an input. If multiple tests were ordered for a given laboratory measure, the first laboratory value available was used as an input to the algorithm. If a specific laboratory test was not performed for the patient, a null value was reported and implicitly handled by the MLA. Inputs were selected based on prior research on mortality in AHF were provided to the 33F XGBoost model during training, including vital sign measurements, laboratory test results, medical history and basic demographic information (Supplementary Table 2). Multiple individual features were derived from each input based on the time of observation, or changes in the observation over time; each of these individual features could then be used by XGBoost as part of classification.

### Gold standard

Mortality was determined by the date of death associated with the unique patient identification codes linked to each patient encounter. If a patient had a death date within 7 days of the initial ED encounter, they were considered to have a mortality event and were included in the positive class. Patients were included in the positive class only if the death date occurred within the selected time window (7 days) for mortality prediction. Patients with mortality dates later than the seven-day window were included in the negative class, as they were still alive within the prediction window. Surviving patients with no documented mortality event were also included in the negative class.

### Machine learning models

Models 33F and Top5F were created using gradient boosting implemented in the XGBoost method in Python [32]. Using this method, results were combined from various decision trees to generate prediction scores. The patient population was split into successively smaller groups within each decision tree, and each tree branch divided patients who entered it into one of two groups according to their covariate value and whether this value fell above or below a determined threshold. The decision tree ended in a set of “leaves” with each AHF patient encounter represented in exactly one leaf. Each patient in a certain “leaf” was predicted to have the same risk of mortality. For comparison, logistic regression and decision tree classifiers were also trained and tested, in order to evaluate the relative utility of the more structurally complex models 33F and Top5F.

A 70:30 training and test split was used to train and evaluate each model’s performance. 70% of AHF patient encounters were randomly selected to train the models, and the remaining 30% were used as a hold-out test set to generate model predictions. We emphasize that, during the training process, XGBoost was not required to use all of the input features in order to make a mortality prediction. Instead, the model could select the subset of features that enabled it to most accurately make classifications based on the training data. Ultimately, this resulted in 33 features being used for the 33F model, despite many more inputs being available, as described in the Data Processing subsection and Supplementary Table 1. The five features with the highest absolute SHAP values for the 33F model were then selected as the input features for the Top5F model and the DT model.

Separately for models 33F and Top5F, a 5-fold cross-validation grid search was conducted on the training set for the purposes of hyperparameter optimization. The optimizations of the hyperparameters for the 33F and Top5F XGBoost models were confirmed by evaluating the AUROC for different combinations of hyperparameters included in the grid search, as demonstrated in Supplementary Table 3. Optimized hyperparameters included maximum tree depth, L1 regularization term (lambda), L2 regularization term (alpha), scale positive weight and number of estimators. By restricting the tree depth to a maximum of 3 branching levels and using a relatively high lambda regularization term we were able to prevent overfitting and increase sensitivity. We also included a constant hyperparameter for the early stopping of the iterative tree-addition procedure, with a value of 5. The only hyperparameter of the DT model that was optimized through a 5-fold cross-validation grid search was the maximum tree depth. The final optimized hyperparameter for the model based on the grid search was 9 for the maximum tree depth. Due to the imbalance of the two classes, the scale positive weight hyperparameter was of particular importance as it determines the rate at which the minority class is oversampled during training. This ensures that as much information as possible is learned about the positive class during training.

For the logistic regression model, first, missing values had to be handled with various imputation strategies. Missing vital signs measurements were imputed using forward filling and backward filling. Missing lab measurements were imputed using the mean measurement of the lab feature from the training set. Then, a 5-fold cross-validation grid search was conducted on the training set for hyperparameter optimization. The only optimized hyperparameter was the strength of regularization. The final optimized value was 1e-5. L2 regularization was used to help prevent overfitting.

After model training, performance was evaluated on a subset of the 30% test set. As a defined list of inputs are required to calculate the EHMRG, evaluating the score’s performance among patients without the required data available would unfairly disadvantage the EHMRG in comparison to the MLAs. To enable a fair comparison between the MLAs and the EHMRG, model performance was evaluated only on test-set patients whose EHR data contained a value for each variable used to calculate the EHMRG, so that the EHMRG could be calculated for all patients in the test-set. These patients comprised 63.2% of the test set. The reported performance metrics are based on the model performance on this subset of the test set. The chosen operating point or threshold is the value above which a patient’s encounter was considered to be positive, or end in the patient’s death (as defined in our gold standard), with performance measured by AUROC. Sensitivity, specificity, positive and negative likelihood ratios, and the diagnostic odds ratios were also evaluated at the clinical operating points for all models. A SHAP (SHapley Additive exPlanations) analysis [13] was performed to evaluate which inputs were most important for the model in generating predictions. A SHAP analysis examines the feature values for each element of the training dataset and evaluates the way in which different values for different features affect the classification of the training examples. The SHAP plot ranks features by importance to model predictions, such that the features which contribute the most to predictions are listed first.

### Comparator

The EHMRG was selected as a comparator for the MLAs [16]. EHMRG also predicts seven-day mortality in AHF patients presenting to the ED and was developed for a comparable use case (i.e., risk stratification to inform hospitalization decisions and clinical management of AHF patients presenting to the ED). In addition, the EHMRG has demonstrated strong discriminatory performance in multiple clinical settings [12, 26, 27, 57], such that it may reasonably be understood to represent the standard of care in mortality prediction for AHF patients in the ED. The following inputs for EHMRG were available within our dataset, and were fed into the comparator model: age, initial systolic blood pressure (SBP), initial heart rate (HR), initial peripheral oxygen saturation (SpO2), first potassium measurement, first creatinine measurement, highest troponin measurement and active cancer diagnosis. Home medication use and method of presentation to the ED were not available in our dataset. Therefore, a complete EHMRG score using previously described thresholds for defining high risk of mortality could not be calculated. Instead, an adjusted EHMRG score was calculated using all available inputs and normalized. To normalize the adjusted EHMRG scores, the maximum and minimum possible EHMRG scores were calculated using the available variables and were then used to rescale adjusted scores between 0 and 1. In order to provide the adjusted EHMRG with the best chance of success in predicting mortality, a new operating point was selected to optimize the adjusted EHMRG’s performance. The operating point on the ROC curve which maximized the square root of the product of true positive rate (sensitivity) and 1-false positive rate (specificity) was selected, for the purpose of balancing demands for sensitivity and specificity. Based on this choice, an adjusted EHMRG score of 49.79, which corresponds to a scaled operating point of 0.364, was selected.

## Supplementary Information

**Additional file 1: Fig. S1.** Machine learning workflow. **Table S1.** Inputs used to train the machine learning algorithm. **Table S2.** International Classification of Diseases, Tenth Revision (ICD-10) codes used to identify emergency department encounters for acute heart failure. **Table S3.** Hyperparameter Optimization for the Top5F and 33F models. For each model, the first row lists the values of the optimal hyperparameters selected during model training. For each subsequent row, a single (bolded) hyperparameter is altered, and all other hyperparameters are unchanged.

## Data Availability

Restrictions apply to the availability of the patient data which were used under license for the current study, and so are not publicly available. The MLA code developed in this study is proprietary and not publicly available.
