# Supplementary material for: Prediction of short-term mortality in acute heart failure patients using minimal electronic health record data
Source: BioData Min. 2021 Mar 31;14:23. doi: 10.1186/s13040-021-00255-w (PMC8010502; doi:10.1186/s13040-021-00255-w)
Supplement: Supplementary file 1 — Additional file 1: Fig. S1. Machine learning workflow. Table S1. Inputs used to train the machine learning algorithm. Table S2. International Classification of Diseases, Tenth Revision (ICD-10) codes used to identify emergency department encounters for acute heart failure. Table S3. Hyperparameter Optimization for the Top5F and 33F models. For each model, the first row lists the values of the optimal hyperparameters selected during model training. For each subsequent row, a single (bolded) hyperparameter is altered, and all other hyperparameters are unchanged. [file 13040_2021_255_MOESM1_ESM.docx]

**Supplementary Materials**

**
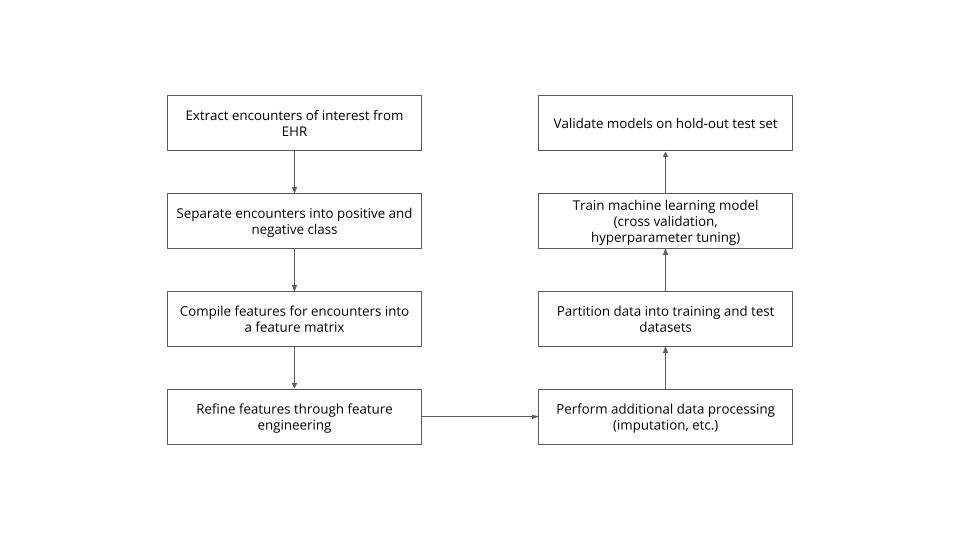
**

**Supplementary Figure 1.** Machine learning workflow.

**Supplementary Table 1.** Inputs used to train the machine learning algorithm.

| **Demographics** | |
| --- | --- |
| Age | Sex |
| **Vital Signs (hourly mean for first 8 hours of ED encounter)** | |
| Systolic blood pressure | Respiratory rate |
| Diastolic blood pressure | Peripheral oxygen saturation (SpO_2_) |
| Mean arterial pressure* | Temperature |
| Heart rate | Hourly change (differential) in all vital signs |
| Fraction inspired oxygen (FiO_2_) |  |
| **Laboratory Results (earliest available)** | |
| Sodium | Glucose |
| Potassium | Hemoglobin |
| pH | White blood cell count |
| Bicarbonate | Lactate |
| Creatinine | Troponin |
| Blood urea nitrogen (BUN) |  |
| **Medical History** | |
| Hypertension | Hepatic cirrhosis |
| Cerebrovascular disease | Cancer |
| Dementia | Previous diagnosis of acute or chronic heart failure |
| Chronic obstructive pulmonary disease (COPD) |  |

*calculated from systolic and diastolic blood pressures

**Supplementary Table 2.** International Classification of Diseases, Tenth Revision (ICD-10) codes used to identify emergency department encounters for acute heart failure.

| ICD-10 | I50.21, I50.23, I50.31, I50.33, I50.41, I50.43, I50.811, I50.813 |
| --- | --- |

**Supplementary Table 3.** Hyperparameter Optimization for the Top5F and 33F models. For each model, the first row lists the values of the optimal hyperparameters selected during model training. For each subsequent row, a single (bolded) hyperparameter is altered, and all other hyperparameters are unchanged.

| **33F Model** | | | | | |
| --- | --- | --- | --- | --- | --- |
| Maximum Tree Depth | L2 Regularization Term (Lambda) | L1 Regularization Term (Alpha) | Scale Positive Weight | Number of Estimators | AUROC |
| 3 | 200 | 1 | 5 | 100 | 0.843 |
| **5** | 200 | 1 | 5 | 100 | 0.814 |
| **7** | 200 | 1 | 5 | 100 | 0.774 |
| 3 | **100** | 1 | 5 | 100 | 0.805 |
| 3 | **150** | 1 | 5 | 100 | 0.810 |
| 3 | 200 | **0.1** | 5 | 100 | 0.811 |
| 3 | 200 | **5** | 5 | 100 | 0.842 |
| 3 | 200 | 1 | **1** | 100 | 0.668 |
| 3 | 200 | 1 | **10** | 100 | 0.816 |
| 3 | 200 | 1 | 5 | **200** | 0.843 |
| **Top5F Model** | | | | | |
| Maximum Tree Depth | L2 Regularization Term (Lambda) | L1 Regularization Term (Alpha) | Scale Positive Weight | Number of Estimators | AUROC |
| 3 | 150 | 0.1 | 10 | 50 | 0.830 |
| **5** | 150 | 0.1 | 10 | 50 | 0.814 |
| **7** | 150 | 0.1 | 10 | 50 | 0.808 |
| 3 | **100** | 0.1 | 10 | 50 | 0.829 |
| 3 | **200** | 0.1 | 10 | 50 | 0.830 |
| 3 | 150 | **1** | 10 | 50 | 0.830 |
| 3 | 150 | **5** | 10 | 50 | 0.830 |
| 3 | 150 | 0.1 | **1** | 50 | 0.629 |
| 3 | 150 | 0.1 | **5** | 50 | 0.824 |
| 3 | 150 | 0.1 | 10 | **100** | 0.830 |
